# Comparative Study of Strain Measurement Techniques for Assessing Creep in CFRP Tendons

**DOI:** 10.3390/s25226897

**Published:** 2025-11-12

**Authors:** Alexandra Boloux, Iurii Burda, Luke A. Bisby, Giovanni Pietro Terrasi

**Affiliations:** 1 Institute of Infrastructure and Environment, University of Edinburgh, Edinburgh EH93JL, UK; luke.bisby@ed.ac.uk; 2 Empa, Swiss Federal Laboratories for Materials Science and Technology, CH-8600 Dübendorf, Switzerland; iurii.burda@empa.ch (I.B.); giovanni.terrasi@empa.ch (G.P.T.)

**Keywords:** CFRP tendons, creep strain, foil strain gauges, digital image correlation (DIC), fiber optic strain sensing, extensometers

## Abstract

The long-term viscoelastic behaviour of epoxy matrices in Carbon Fibre-Reinforced Polymer (CFRP) tendons can lead to creep strains which must be accurately quantified to improve the current necessarily conservative design guidelines for bridge applications. However, the task of experimentally capturing such strains—typically in the range of 0.05%—requires sensors with reliable long-term accuracy and precision. This study investigates creep in CFRP tendons subjected to sustained tensile loading at 80% (for 7 days) and 88% (for 22 h) of their ultimate tensile strength. Four strain sensing techniques were employed to capture the creep strains of the CFRP tendons: bonded metal foil strain gauges, a contact extensometer, Digital Image Correlation, and distributed fibre optic strain sensing. This work precisely quantifies—for the first time in CFRP creep testing—the influence of experimental artefacts on the performance of the strain sensors, including test rig movement, temperature sensitivity, and localised surface inhomogeneities. Results reveal significant measurement distortions: the extensometer recorded strain increases of 250% during tendon slip, while distributed fibre optics detected localised strain peaks reaching 150% of the surface average. These findings demonstrate that sensor-induced noise can substantially contaminate creep strain data, underscoring the critical need for rigorous experimental protocols and thorough sensor validation in CFRP creep studies.

## 1. Introduction

Robust experimental research depends on reliable measurements of key variables of interest. As materials and products in the building industry evolve, often into stronger, stiffer, and more durable materials, sensor technology can become a limiting factor in reliably assessing material mechanical performance. One notable example is Carbon Fibre-Reinforced Polymer (CFRP) tendons, which are increasingly being used in bridge applications for prestressing and hangar cables. The strength-to-weight ratio of CFRP tendons or cables can be 15 times higher than those made from traditional high tensile strength steel. In the context of long-term tensile testing under high sustained loads, i.e., creep testing, the improved material properties of CFRPs can present challenges for discerning differences in mechanical response using a range of strain measurement technologies.

The significant potential of CFRPs to improve material, product, system, and structural efficiencies is presently limited by necessarily conservative design guidelines. For instance, the American Concrete Institute suggests a tensile in-service stress limit of 0.55 of the characteristic tensile strength of unidirectional CFRPs [1]; European standards recommend a similar limit of 0.51 for unidirectional CFRP bars and tendons [2]. These limitations are partly justified by the viscoelastic behaviour of CFRPs. Over time, under sustained loading, thermosetting polymer-matrix materials may develop creep strains which, if they become significant, could introduce potential structural deformations and eventually failure mechanisms (specific to their application). It is therefore important to accurately quantify the long-term creep strain responses in CFRP tensile elements using reliable test set-ups and strain sensors with sufficient long-term accuracy and precision.

This paper focuses on the latter issue and compares the performance of various strain sensing techniques under sustained loading. Most existing studies on creep of CFRP composites have relied on bonded foil strain gauges to quantify strains [3,4,5]. For instance, Ando [6] reported using a ′pi-gauge′ which consisted of strain gauges bonded on the inner and outer curvatures of an omega-shaped steel gauge, which was itself mechanically fixed to CFRP samples—albeit a description of the attachment is not provided. Jiang et al. [7] used a high-temperature contact extensometer to measure creep strains of CFRP coupons at elevated temperatures. Yang et al. [8] developed a custom dial gauge device which was ′validated′ by comparison against bonded foil strain gauges. Meanwhile, Anderegg et al. [9] reported on 17 years of strain monitoring of CFRP tendons, in three bridges in Switzerland, using adhesively bonded foil strain gauges and Fibre Bragg grating optical fibres that had been embedded within the CFRP wires during the pultrusion process.

While Anderegg’s extensive field work appears to show long-term stability of the strain sensing equipment at comparatively low CFRP sustained load levels of 45% of the ultimate tensile strength (UTS), it also highlights that annual temperature variations of 45 °C induce strain variations of 25% of the initial pretension strain for both the bonded strain gauges and the embedded optical fibres. The sensitivity of bonded strain gauges to temperature is well established in the literature, and many data acquisition systems can automatically correct strain data for temperature effects [5]. Moreover, Zhao et al. [10] warn of the sensitivity of adhesively bonded foil strain gauges to creep, and the serious and complex non-linear errors that the use of such sensors can generate during creep experiments. The creep characteristics in bonded foil strain gauges are partly related to a relaxation of the adhesive between their foil grids and the polymer backing sheet, but also to the loading and environmental conditions. As an example, Tuttle et al. [11] measured strains in graphite–epoxy composite laminates, loaded at 60% of their UTS, with adhesively bonded foil gauges, and compared experimental results to theoretical predictions derived from the Schapery nonlinear-viscoelastic theory: when the loading time was less than 10,000 min, predicted results were consistent with experimental data, but for loading times greater than 10,000 min (about 6.9 days), the two data sets diverged, with differences as high as 10%.

In typical short term tensile testing, thermal and creep strains of the sensors are not a serious concern due to the short test duration and essentially uniform temperature, unlike in creep tests during which the composite may be subject to prolonged test durations (generally 1000 to 3000 h), and held at high sustained strains and with varying temperatures. For example, Phoeuk et al. [4] reported strain levels of over 18,000 με for pultruded CFRP grids during a 10,000 h creep test at 92% of the grids’ UTS. Moreover, current creep studies report extremely small creep strains for CFRP composites. Yang et al. [8] recorded 150 με for pultruded CFRP tendons loaded at 85% of UTS for 1000 h; Jiang et al. [7] measured 573 με for pultruded CFRP tendons loaded at 60% of UTS for only 4 h; Ascione et al. [5] measured 50 με for pultruded CFRP laminates loaded at 75% of UTS after 1824 h. In such cases, the magnitude of the strains caused by thermal sensitivities and creep of the strain sensors themselves can become significant, making accurate quantification of creep performance challenging. Despite the high level of precision required for measuring creep strains of CFRP composites, there is a lack of clarity on data variability and possible measurement noise processing in current creep studies on CFRP. Yang et al. [8] have mentioned fluctuations in their strain data and attributed these to measurement errors and thermal deformations of the testing device, but have not quantified the magnitudes of those fluctuations, nor deeply investigated their causes.

This paper provides a comparative assessment of different strain measurement techniques to quantitively describe their performance under high sustained stresses and assess their reliability for measuring creep in CFRP tendons over long durations. In particular, this study focuses on three different typical adhesively bonded foil gauges, a distributed fibre optic sensing (DFOS) system, a contact extensometer, and Digital Image Correlation (DIC). The main objectives of this study are to identify sources of noise in long-term creep studies of CFRP tendons at high loads and precisely quantify their influence on the measured strain data to separate sensor-induced effects from the true material behaviour of the CFRP tendon. For the first time in the context of CFRP tendon creep studies, this research systematically evaluates how measurement artefacts affect strain data, enabling more accurate interpretation of material behaviour.

After introducing the CFRP materials used and various strain and environmental sensors tested, the main sources of noise in the unprocessed experimental data are identified and their effects on the measured overall creep strain of the CFRP tendons are precisely quantified. Finally, a comparison of the creep strain measurement results for the cross-validated strain sensing techniques is provided, establishing best practices for long-term creep monitoring of CFRP tendons.

## 2. Materials and Methods

### 2.1. CFRP Tendons

The tendons in this study are CFRP bars nominally 5.08 mm in diameter. They were pultruded between 1995 and 1997 by Stesalit Ltd. (Zullwil, Switzerland), using Toray T700 carbon fibres (Toray Industries Inc., Tokyo, Japan) embedded in LY556/HY917/DY070 epoxy matrix (Ciba-Geigy AG. now Huntsman, Bad Säckingen, Germany). The tendons were stored indoors in a controlled laboratory environment at the Swiss Federal Laboratories for Materials Science and Technology (Empa, Dübendorf, Switzerland) since their production. The fibre volume amount is 72%, determined by thermogravimetric analysis. The coefficient of thermal expansion (CTE) of the fibres is −3.8 × 10^−7^/°C [12], while for the matrix it is 56.0 × 10^−6^/°C [13]. Assuming that the fibres and the matrix have the same strain in the axial direction, and neglecting transverse stresses associated with thermal expansion of both the fibres and matrix, the CTE for the composite in the longitudinal direction can be estimated from the rule of mixtures [14]:

(1) $$a_{11}=\frac{\Phi_{F}E_{F}\alpha_{F}+\alpha_{M}E_{M}\left(1-\Phi_{F}\right)}{\Phi_{F}E_{F}+E_{M}\left(1-\Phi_{F}\right)}$$

where $\Phi_{F}$ is the fibre volume amount, $E_{F}$ and $E_{M}$ are the elastic modulus values for the fibres and the matrix, respectively, and $\alpha_{F}$ and $\alpha_{M}$ are the CTEs for the fibres (in the direction of the fibres) and the matrix, respectively. Given elastic modulus values of 230 GPa and 3.3 GPa for the fibres and the matrix correspondingly, $a_{11}$ was calculated as −6.7 × 10^−8^/°C.

The CFRP tendons have a UTS of 3035 MPa and an elastic modulus of 156.6 GPa (secant modulus between strain levels of 0.0% and 0.5%), measured by distributed fibre optics in a preliminary study of the residual tensile properties of the same CFRP tendons [15]. The three specimens fitted with distributed fibre optics averaged a strain at failure of 1.86%.

### 2.2. Strain Sensors

#### 2.2.1. Foil Gauges

Adhesively bonded linear foil gauges were selected from two well-established strain sensing companies: the German manufacturer HBK (Hottinger Brüel et. Kjaer GmbH, Darmstadt, Germany), and the Japanese manufacturer TML (Tokyo Measuring Instruments Laboratory Co., Ltd., Tokyo, Japan) The strain gauges presented in this study were recommended for high performance strain measurements; however, according to specifications, both manufacturers warned that bonded strain gauges, in general, may not be reliable under high sustained stresses due to the creep of the gauge itself [16,17]. Relevant dimensions and properties for each foil strain gauge are presented in Table 1. The three selected gauges all have equal gauge length (‘a’) and similarly small gauge widths (‘b’) to facilitate application on a rounded surface and reduce transverse strains on the bonded foil gauge upon loading. The gauge factor of the strain gauge is the ratio of the relative change in electrical resistance to the mechanical strain and is thus a measure of the sensitivity of the bonded foil gauge to strain.

In addition, three similar cold-curing adhesives were used in this study to bond the foil gauge to the surface of the tendon:

- HBK’s methacrylate X60;
- HBK’s cyanoacrylate Z70, and its newer version CA80;
- TML’s EB2, recommended by the manufacturer for long-term measurements [17].

Consequently, four foil gauge systems, i.e., combinations of foil gauge and adhesive, were established as shown in Table 2.

The glueing of the foil gauges on the surfaces of the CFRP samples was conducted by the same experienced technician at Empa for all samples to reduce variability in the application process. Four lead wires, two excitation and two signal wires, were soldered to the connecting terminals of the foil gauges and connected to the loading frame acquisition system, thus synchronising load and strain measurements. The four-wire circuit used in this study increases the accuracy of the strain signal measured by the strain gauges by compensating for any resistance or temperature-induced changes in the lead wires. The HBK gauges were cured for 1 min using a custom-made clamp for repeatability, while for the TML gauges, a specific gauge mate (type TML GMR-S) was used to apply a uniform pressure of 0.2 MPa for 24 h.

#### 2.2.2. Contact Extensometer

The multiXtens is an in-built, fully automatic contact extensometer coupled to the Zwick loading frame used in this study. During testing, knife-edge sensor arms were clamped onto the surface of the specimen with a maximum clamping force of 2.5 N at a set gauge length of 250 mm and the travel distance of the sensor arms from the original position was measured. The strain of the sample was then computed as the ratio of the travel distance over the initial distance. Measurement accuracy was within 1 μm, over a travel range of 700 mm with an accuracy class of 0.5 according to ISO 9513 [19]. No information is available from the manufacturer on the temperature sensitivity of the multiXtens extensometer.

#### 2.2.3. Digital Image Correlation (DIC)

**Figure 1:**
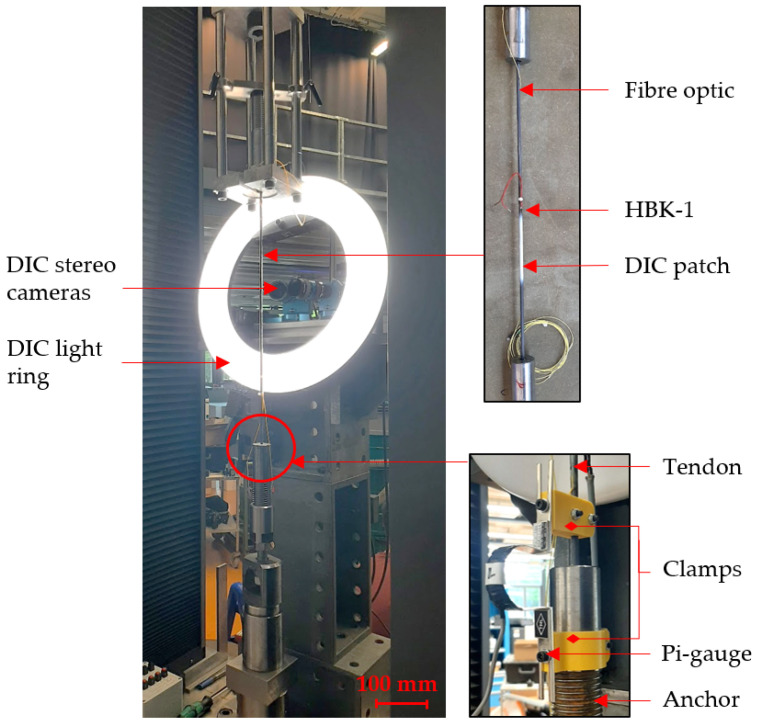
Test set-up for Series A.

DIC is an optical, non-contact strain measuring technique based on the tracking of speckles on the surface of the material. In this study, the Q-400 system developed by Dantec Dynamics GMbH (Ulm, Denmark) was used. Images were recorded by 2 LIMESS (Messtechnik & Software GmbH, Krefeld, Germany) stereo cameras using LINOS MeVis 50 mm f/1.8 lenses with a resolution of 2752 × 2206 pixels (about 8 μm/pixel) to enable accurate measurements on the rounded surface of the tendon and capture potential out-of-plane motion. Before each test, the region of interest on the sample was cleaned with ethanol and coated with a high contrast random speckle pattern using a spray paint gun: first, a uniformly flat layer of white paint was applied, air dried, then sprayed with black paint to create a high contrast speckle pattern (see Figure 1).

**Figure 2:**
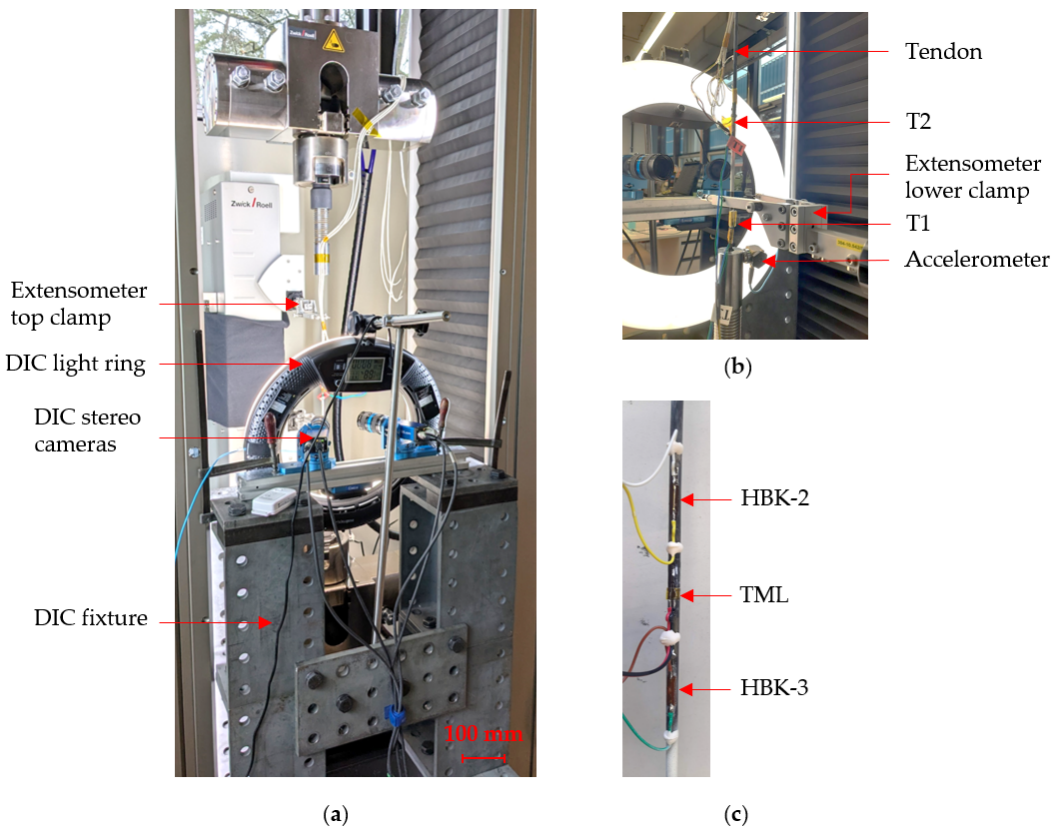
Test set-up for Series B: (**a**) front view; (**b**) back view; (**c**) strain gauge arrangement.

The cameras were securely fixed to the loading frame with a custom steel rig to minimise movement of the cameras relative to the loading frame, as shown in Figure 1 and Figure 2a. A ring light was also attached to the rig for uniform brightness of the DIC speckle patch. Image acquisition and evaluation was conducted with the Istra4D software (version 4.6, Dantec Dynamics). Before and after each test, a calibration was performed to determine the geometric properties of the camera set-up, such as the angle between the two cameras and their distance to the sample. During the loading phase, the image acquisition rate was set to 1 Hz, and subsequently decreased, depending on the test duration, during the sustained loading phase.

Image evaluation was conducted using a virtual gauge length approach. As such, two vertically aligned points were selected on the surface of the tendon and a square area of 29 by 29 pixels, called the facet, was defined around the selected point to contain 3–5 distinct black speckle dots. The distinct speckle pattern was then input in the software’s search algorithm to find the facet with the identical same speckle pattern on all subsequent images. Given known calibration parameters, the position of the start and end point of the gauge length, at each time step, was translated to a set of xyz coordinates. The vertical distance between the two end points on a same image was then calculated and the strain was computed as the ratio of the change in distance over the initial distance. This approach was adopted to bypass analytical software assumptions and mathematical approximations required to compute a distributed strain map. This approach also enables a fairer comparison with the contact extensometer and the bonded foil gauges, since both strain sensing techniques use a set gauge length to measure strains of the CFRP tendons.

#### 2.2.4. Distributed Fibre Optic Sensing (DFOS)

A comparatively novel technique of strain measurement is the use of DFOS to measure strains along the free length of the tendon. In these tests, a single optical fibre of type Fibercore SM1250B3(9.8/125) (Humanetics, Southampton, UK) was bonded to the surface of the tendon with rapid curing epoxy glue (Suter Kunststoffe AG, Fraubrunnen, Switzerland). The optical fibre has a core diameter of 9.8 μm, a cladding diameter of 125 ± 1 μm, and a coating diameter of 155 ± 5 μm [20]. The fibre was connected to a high-definition fibre optic sensing system, the LUNA ODiSI 6104 system (LUNA, Roanoke, VA, USA), working on the principle of Rayleigh backscatter [21]. This system measures changes in the frequency of backscattered light from a laser source through the fibre core when the fibre is compressed or elongated, as occurs when the tendon is subject to mechanical strain. Recent studies at Empa have shown the capabilities of the LUNA system in various CFRP applications and geometries [22,23]. Strains were continuously monitored along the middle 300 mm of the tendon’s free length, and at a distance interval of 0.65 mm. The rate of acquisition of the DFOS was set at 0.2 Hz. The strain signal was manually synchronised with the loading. Due to the limited service life of the laser source (typically 25,000 h in a LUNA ODiSI DFOS interrogator) and high replacement cost (approximately GBP 25,000), continuous measurements were limited to 20 h in duration. Furthermore, the refractive index of the fibre is temperature dependant which can lead to temperature-induced apparent strain variations estimated to be in the order of 10 με/°C.

### 2.3. Other Sensors

Temperature was measured directly at the surface of the tendon with 2 resistance temperature detectors (RTDs), type PT100, named T1 and T2. T1 was taped at the mid-section of the tendon, behind the bonded strain gauges, while T2 was taped closer to the bottom anchor, under the bottom clamp of the contact extensometer, as shown in Figure 2b. In both cases, the backing of the temperature detectors was coated with thermal paste to improve the heat transfer between the tendon’s surface and the temperature sensor. The two temperature detectors were connected and synchronised to the Zwick data logger. In addition, an ambient thermo-hygrometer, type Sensirion SHT40 (Sensirion Holding AG, Stäfa, Switzerland), was used to record back-up values for ambient temperature and relative humidity. In the second test series, the two RTDs recorded similar temperature variations which closely matched the temperature data measured by the Sensirion detector. As such, only temperature data recorded by T2 is used in the following sections.

Vibrations of the testing set-up were recorded with a PCB Triaxial Accelerometer (National Instruments, Austin, TX, USA). A custom algorithm was used to enable long-term measurements and data saving: vibration data was read into a buffer at a frequency of 1000 Hz, the peak in vibration was then identified and saved for every minute of testing, while the rest of the buffer was cleared.

Finally, a pi-gauge displacement transducer, type PI-5-100 (Tokyo Measuring Instruments Laboratory Co., Ltd., Tokyo, Japan), was attached to the tendon to measure slip out of the loaded anchor (see Figure 1). This sensor is a combination of two TML FLAB-5-11 linear foil gauges attached to the outer and inner curvature of a flexible steel arch-shaped spring plate. PLA (Polylactic Acid) plastic 3D-printed clamps mechanically secured the pi-gauge over the bottom anchor and on the tendon.

### 2.4. Test Set-Up

Before testing, each tendon was anchored in steel barrels filled with epoxy resin, type L20/EPH 161, as described elsewhere [15]. After casting and curing of the anchors, a graphite powder was sprayed on the hardened epoxy cone to reduce friction between the barrel and the resin cone during loading. Each tendon was pre-loaded to 30 kN to set the resin cone in the barrel and reduce the risk of tendon pull-out during loading. Foil gauges and/or fibre optics were then bonded to the surface of the tendon, and DIC speckle patterns were applied.

#### 2.4.1. Series A

The aim of the first testing series was to compare the performance of the HBK-1 foil gauge, the extensometer, the DIC, and the DFOS under high sustained strains. Tests were conducted in June 2024 at Empa. In this set-up, three tendons were loaded in tension in a Walter & Bai loading frame (Walter & Bai, Löhningen, Switzerland), at a rate of 4 mm/min. The tendons were loaded to 50 kN, which corresponds to 80% of their UTS, and a strain of 1.42% calculated from the known value of elastic modulus of the CFRP tendon. The tendons were fitted with a bonded HBK-1 foil gauge, DIC, one bonded fibre optic, and a clamped pi-gauge, as seen in Figure 1. All measurements were manually synchronised at the start of loading. During the sustained loading phase, DIC images were acquired every 5 min for the first 2 h, then every 30 min until the end of the test. To achieve the frequency of 30 min, an external waveform generator type 33120A (Agilent, Santa Clara, CA, USA) was coupled to the Dantec system and a step function was used to trigger image capture. The test duration was set as 7 days under sustained loading. However in Test A2, due to a power cut, the test was stopped after 10 h; the exact same tendon was then reloaded for another 7 days. Both measurements cycles are presented in the Section 3. In Test A3, during the initial 7 days of loading, it was observed that the foil gauge signal was faulty due to a bad connection, hence the exact same tendon (with the initial glued foil gauge) was reloaded for another 7 days to record a valid signal from the foil gauge. Unfortunately, the DIC image capture system suffered from irregular but unavoidable interruptions in the data acquisition, potentially caused by a data buffer overload.

#### 2.4.2. Series B

A second series of tests was conducted in February 2025 at Empa to assess the behaviour of three bonded strain gauge systems (HBK-2, HBK-3, TML), the multiXtens contact extensometer and DIC. Due to the lack of free surface on the tendon’s outer diameter, already equipped with three bonded foil gauges, a DIC paint patch and the clamps of the contact extensometer, it was not physically possible to glue an additional fibre optic. Three tendons were loaded in tension in a ZwickRoell Z250 loading frame (ZwickRoell, Ulm, Germany) as shown in Figure 2. The tendons were loaded at a rate of 4 mm/min to 54 kN, which corresponds to 88% of their UTS, and a calculated expected strain of 1.53%. All measurements were manually synchronised with the start of loading. The duration of loading was defined from the first series of tests which showed that a duration of 20 h was sufficient to develop the expected creep strain curve profile. One tendon failed after 4.5 h of sustained loading, due to the anchorage system failing; the other two tendons sustained loading for 20 h before the tests were terminated.

Temperature measurements, strains from the foil gauges, and strains from the extensometer were acquired at 1 Hz. In the first two tests, measurements from the loading frame (force, crosshead displacement, and strain from the extensometer) were acquired at a frequency of 2 Hz. This caused an unforeseen overload of the data acquisition system, which stopped acquiring data after 16 h (except for a few data points just before unloading the tendon). Consequently, in the last test, the frequency was set as 1 Hz. DIC images were captured every minute during the first two hours of sustained loading, then every 10 min until the end of the test.

Table 3 summarises the testing matrix and introduces the test nomenclature used in the following sections:

## 3. Results and Observations

Four main sources of variability in the measured strain data are highlighted: movement and creep of the test set-up, limitations of strain gauges, sensitivity of strain sensors to temperature, and material inhomogeneity. In both Test Series, A and B, the force in the loading frames was monitored and found to be constant within 0.005% in the Zwick and 0.025% in the Walter & Bai, of the set sustained load. Force variability should naturally be the first source of concern when assessing potential variability in sustained strain data, and minimised wherever possible.

### 3.1. Mobility and Creep of the Test Set-Up

#### 3.1.1. Extensometer Discontinuities

**Figure 3:**
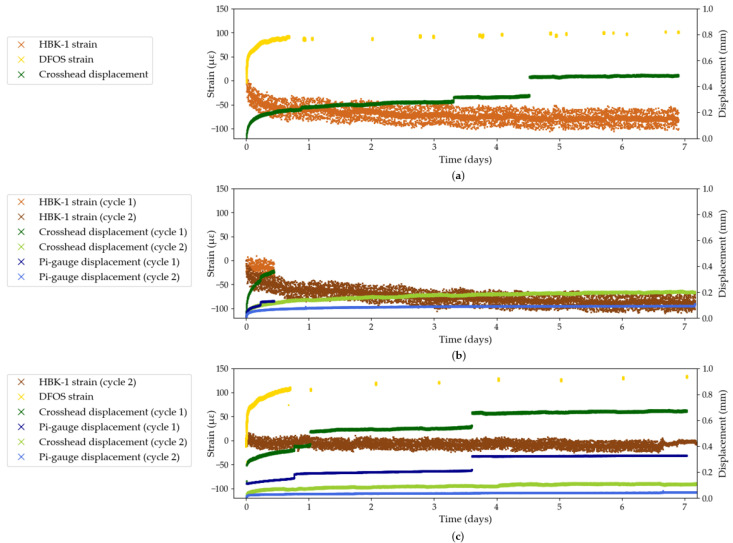
Strain and displacement data from Test Series A: (**a**) Test A1; (**b**) Test A2; (**c**) Test A3.

Strain measuring techniques are, by definition, sensitive to displacements. As such they will capture displacements of the sample but also potentially movements of the test set-up. Figure 3 shows the results (zeroed from the start of sustained loading) of Test Series A for all strain sensing techniques except DIC, which is discussed in Section 3.4.2. Strain measurements from the HBK foil gauge and the fibre optic can be read on the primary y-axis (on the left); a negative value indicates a decrease in strain from the set stress level. Displacement measurements from the crosshead and the pi-gauge are read on the secondary y-axis (on the right). In Tests A2 and A3, both cycles are presented. No smoothing has been applied to the data.

It can be seen in Tests A1 and A3 that the crosshead displacement curve from the first cycle exhibits sporadic jumps of about 0.1 mm. These jumps, which were generally accompanied by a sharp noise, are thought to be caused by the setting of the resin cone in the anchor head, or by the pull-out of the tendon from the resin cone. Displacements measured with the pi-gauge corroborate this hypothesis: the jumps in crosshead displacement in Test A3 are synchronised with jumps in the pi-gauge displacement recordings and are in the same order of magnitude. This observation is true for first cycle measurements. However, it can be observed in Test A3 that the crosshead and pi-gauge displacement curves during the second loading cycle are smooth, and increase at a lower rate than during the first cycle. In fact, the resin cone is likely fully wedged in the steel anchor after the first cycle, which leads to a stiffening of the testing set-up. It is noteworthy that this is also the case in Test A2, despite the first cycle only lasting 10 h, during which no major jumps in displacement were recorded either by the crosshead displacement or by the pi-gauge.

The residual increase in displacement measured by the crosshead and the pi-gauge in the second cycle in Tests A2 and A3 can be attributed to creep of the resin cone in the anchor barrel. The potted anchor is indeed a significant variable in the reliability of the long-term test set-up because it creates a state of localised transverse compressive stress around the potted tendon, due to the stiffness of the epoxy resin, as shown in a study by Züst [22]. During long-term sustained stress testing, creep of the potting resin may induce changes in the stiffness of the epoxy resin, which may heighten localised transverse stresses around the potted tendon. This may, in turn, lead to a local increase in apparent measured creep strains of the anchored CFRP tendon. As such, and despite the great challenge this task presents, creep of the resin cone should not be confused with creep of the tendon itself and efforts should be undertaken, where possible, to separate the two phenomena during analysis.

Both bonded strain sensing techniques, the HBK foil gauges and the DFOS, do not experience jumps in strain and are thought to not be affected by the creep of the anchors. Indeed they provide a strain measurement at a certain distance from the end anchors. It can, however, be observed, most visibly in Test A1, that the strain data recorded by the HBK-1 foil gauge presents a uniform level of noise, in the order of ±25 με, despite an apparent concentration of data at the mean value. On the other hand, the strain measurement from the DFOS appears to be significantly less noisy in the first 20 h of continuous measurement, compared to the strain data from the HBK-1 foil gauge.

**Figure 4:**
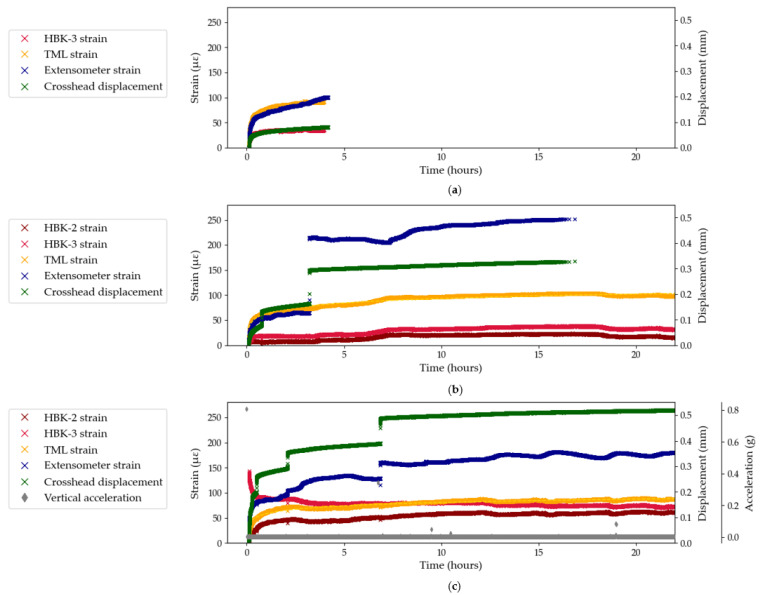
Strain and displacement data from Test Series B: (**a**) Test B1; (**b**) Test B2; (**c**) Test B3.

Figure 4 shows the results from Test Series B, which investigates the performance of three bonded foil gauge systems and the contact extensometer. Similarly to Test Series A, the crosshead displacement exhibits sporadic jumps in displacement which do not lead to jumps in the strain measured by the bonded strain gauges. On the other hand, the extensometer experiences the same jumps as the crosshead, both in synchronicity and magnitude. Indeed, the extensometer clamps are not bonded to the surface of the tendon and a sudden displacement likely causes the clamps to slip and hence record a discrete (and false) increase in measured strain. For example, in Test B2, the jump in crosshead displacement in the third hour of testing causes the extensometer to record a strain increase of 150 με which represents a 250% increase from the strain measured before the jump. This represents a testing artefact which should be eliminated during data processing. This testing artefact is corroborated by the visible jumps in acceleration readings in Test B3, which match the jumps in crosshead and extensometer displacement, thus confirming the overall movement of the anchored tendon as opposed to intrinsic material deformation in the CFRP composite. This observation also stresses the importance of continuous measurements which are able to capture sudden changes in measured strain during long-term sustained loading tests.

#### 3.1.2. DIC Pre- and Post-Test Calibration

DIC measurements are particularly affected by the movement of the test set-up. Indeed, this technique relies on spatial tracking of pixels from a reference step and any movement of the DIC cameras or testing set-up between the reference step and a step in time may lead to erroneous strain measurements. In both Test Series, see Figure 1 and Figure 2, the DIC cameras were levelled and attached to heavy steel blocks which rested on the immobile base plate of the loading frame, thus maintaining free movement of the DIC set-up relative to the loading frame while minimising movement of the cameras relative to the frame caused by external vibrations such as the potential vibrations following nearby test failures. Nevertheless, as previously demonstrated in Section 3.1.1, with the jumps in extensometer displacement, movement can originate within the loading frame itself, for example, when the tendon suddenly slightly slips out of the anchor.

**Figure 5:**
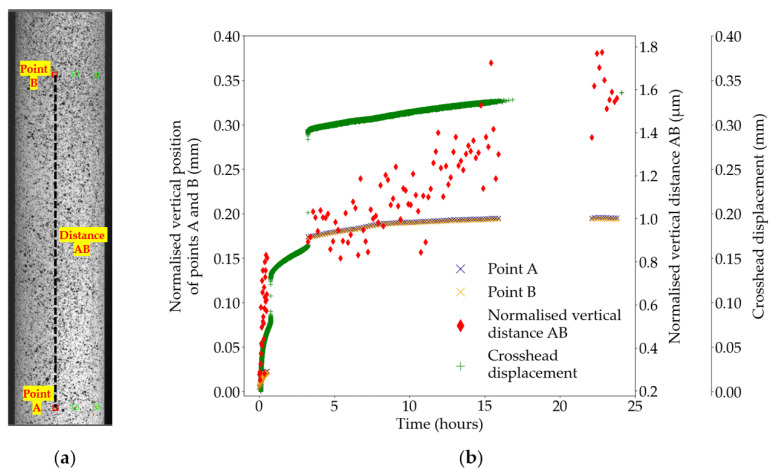
Test B2, DIC displacements; (**a**) position of the points A and B on the region of interest; (**b**) displacement data for points A and B, distance AB, and the crosshead. The lack of DIC data between hours 1–3 and hours 16–23 corresponds to interruptions in the DIC image capture system.

Figure 5 shows the tracking by DIC of the position of two vertically aligned points A and B on the Region of Interest (ROI) of the sample in Test B2. As shown in Figure 5b, the two points experience a shift in their vertical position, normalised to their respective starting position, between the first and the fourth hour of testing. More importantly, both points move by the same distance of 0.15 mm, which corresponds to the cumulative distance of the jumps in crosshead displacement at the first and the third hour of testing. Consequently, the vertical distance between points A and B maintains a gradual increase in magnitude throughout the test, independent of the jumps in crosshead displacement. For clarity, only one data set is presented in Figure 5; however, this validation check has been applied to all DIC data sets before computation of strains.

**Figure 6:**
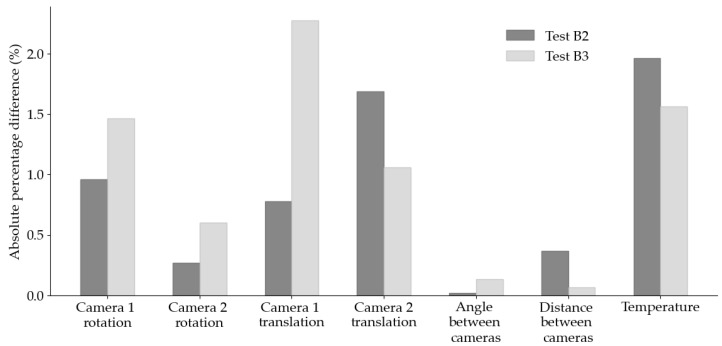
Absolute percentage differences of calibration parameters between pre-test and post-test calibration for Tests B2 and B3.

To further investigate the influence of movement of the test set-up on the measured DIC strain data, the calibration parameters of the DIC set-up were obtained pre-test and post-test. The absolute percentage difference between pre-test and post-test values for certain calibration parameters of interest, are presented in Figure 6 for Tests B2 and B3. In particular, the rotation and translation vectors for each individual camera, the angle and the distances between both cameras, and the camera temperatures are evaluated. In general, the difference between pre-test and post-test values is less than 2%, except for the translation vector for camera 1 in Test B3 which differs by 2.3%; however, this greater difference does not lead to an increase in difference in the angle or the distance between both cameras. Indeed, the smallest differences occur with the two calibration parameters which assess the geometry, in terms of angle and distance, of both cameras as a system. For example, the angle between both cameras differed by 0.01 degrees during Test B2 and 0.06 during Test B3. Despite these small differences, the change in angle between the cameras may alter the point coordinates found during evaluation; hence, the gauge lengths are determined and the strains are calculated.

**Figure 7:**
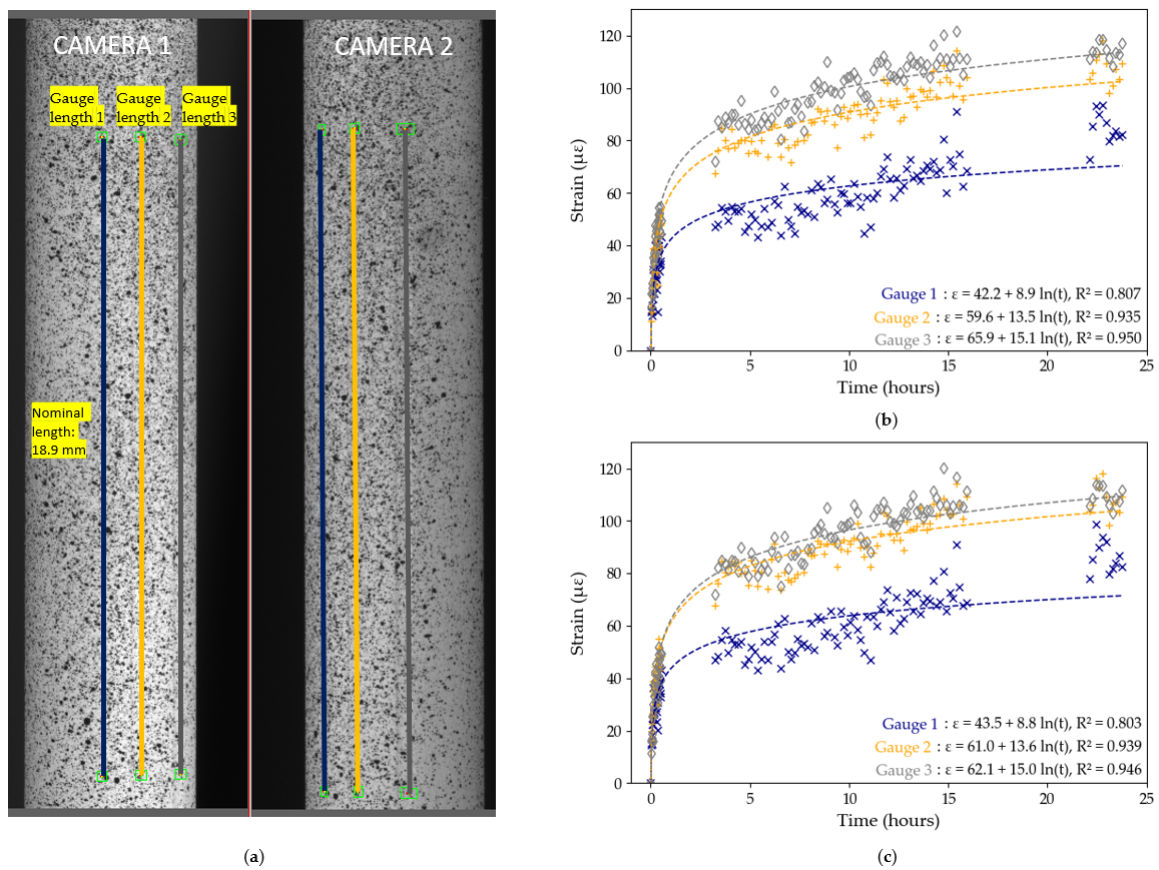
Test B2, DIC results: (**a**) position of the gauge length on the region of interest; (**b**) strain data using pre-test calibration parameters with logarithmic trendlines; (**c**) strain data using post-test calibration parameters with logarithmic trendlines. The lack of data between hours 1–3 and hours 16–23 corresponds to interruptions in the DIC image capture system.

**Figure 8:**
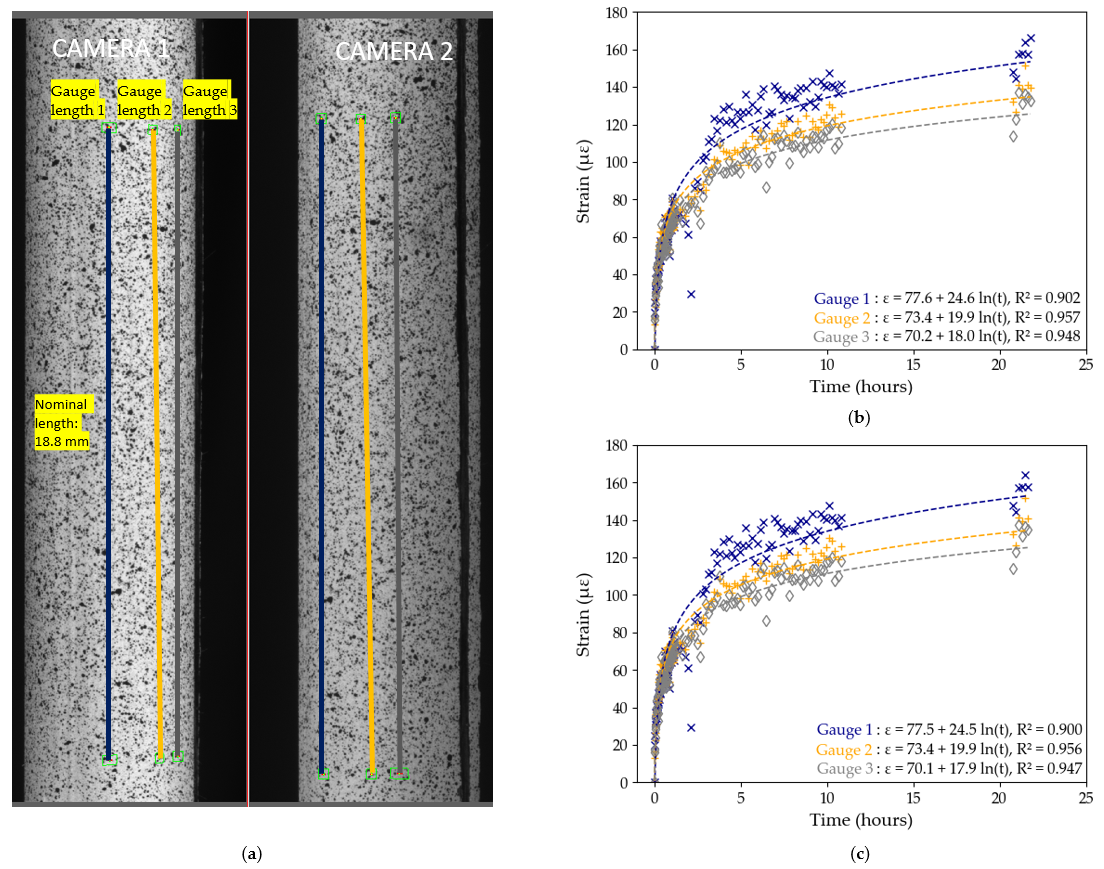
Test B3, DIC results: (**a**) position of the gauge length on the region of interest; (**b**) strain data using pre-test calibration parameters with logarithmic trendlines; (**c**) strain data using post-test calibration parameters with logarithmic trendlines. The lack of data between hours 11–21 corresponds to interruptions in the DIC image capture system.

The influence of pre-test and post-test calibration parameters on the measured DIC strains is investigated in Figure 7 and Figure 8 for Tests B2 and B3, respectively. In each test, three gauge distances, of comparable length, are tracked across the diameter of the tendon, as shown in Figure 7a and Figure 8a on both cameras. The strain data for each of the three gauges is then computed, first using pre-test calibration parameters, then using post-test parameters. The results from Figure 7 and Figure 8 show variability in strain data between the different gauge length position within a same test. For example, in Test B2, with pre-test calibration parameters (see Figure 7b), the strain recorded by gauge length 3 is 61% higher than the strain recorded by gauge length 1, despite a distance between both gauges of only 2 mm across. These findings contradict with the expected uniform longitudinal strain distribution in unidirectional CFRP tendons which highlights that the differences in strain between the gauge lengths is an experimental DIC artefact rather than an indication of the behaviour of the CFRP sample. The sensitivity of the DIC measuring technique to the steep curvature of the CFRP tendon thus has a significant influence on the reported measured strains of the tendon. Moreover, logarithmic trendlines, which were shown to accurately describe long-term strain data of CFRP tendons [8], were fitted to each set of *x*-*y* data:

(2) y=aln(x)+b

where parameters *a* and *b* were found from least squares regression analysis performed using Python 3.11.

The equations for each line are shown in Figure 7 and Figure 8, with the $R^{2}$ value, which represents the degree of fit of the data to the logarithmic trendline. In general, in both tests and in both the pre-test and post-test evaluation, the DIC strain data exhibit a high degree of precision as shown by the high $R^{2}$ values. In Test B2, the maximum difference in $R^{2}$ values between pre-test and post-test evaluation is only 0.004, while in Test B3 it is only 0.002. In general, however, gauges 2 and 3 have higher $R^{2}$ values and closer matched trendlines compared to gauge 1. Based on these findings, we decided to use Gauge Length 3 with pre-test calibration parameters in Test B2, and Gauge Length 2 with pre-test calibration parameters in Test B3 in subsequent data analysis, since these data sets had the highest $R^{2}$ values.

The logarithmic equations from Figure 7 and Figure 8 can be used to further quantify the degree of influence of the movement of DIC cameras on the overall measured strains by extrapolating the strains predicted to occur after 50 years (48,000 h): in Test B2, using Gauge 3, the 50 year strain prediction is 262 με (pre-test calibration) and 257 με (post-test calibration); in Test B3, using Gauge 2, it is 332 με (pre-test and post-test calibration). The maximum difference of 10 με after 50 years, obtained in Test B2, between pre-test and post-test calibration parameters, is insignificant in the context of civil engineering applications.

On account of these findings, the influence of movement of the test set-up on DIC data is considered to be minimal and the results from DIC evaluation in Tests B2 and B3 are deemed robust. This sensitivity study nevertheless highlights the need for a stable DIC set-up during testing and a thorough data analysis to minimise testing artefacts.

### 3.2. Limitations of Strain Gauges

In this section, the performance of the different foil gauge systems is explicitly investigated. Firstly, in the three tests from Series A (Figure 3), the HBK foil gauges measured a decrease in strains which contradicts with the expected theoretical behaviour of the CFRP tendon in creep under sustained loading. The performance of the HBK foil gauges under sustained loading is further investigated in Test Series B and compared to the performance of the TML foil gauges (Figure 4). In Test B1, which only shows the first four hours of sustained loading (due to anchor-induced failure of the sample), the strain profile measured by the TML gauge matches that measured by the contact extensometer: as such, a creep strain of about 100 με was achieved after four hours. On the other hand, the strains measured by the HBK gauge were stagnating at 35 με. Similarly in Test B2, the HBK-2 and HBK-3 systems did not exceed 35 με whereas the TML system recorded strains of 100 με. Meanwhile, the contact extensometer recorded maximum strains of 126 με (after removing the sudden jump in strain), which seems to confirm the inaccurate strain measurements by the HBK-2 and HBK-3 foil gauge systems. Finally, in Test B3, the TML foil gauge recorded a maximum strain of 86 με which is lower than the strain measured by the extensometer (156 με after removing the jumps in strain). However the variability in the signal recorded by the extensometer (which will be discussed in the next section) questions the accuracy of the extensometer measurement in this test. The HBK-2 system records a maximum strain of 60 με which is an increase compared to the previous test but remains an underestimation compared to the strains recorded by the TML foil gauge. HBK-3 exhibits an instantaneous decrease in strain: this is not a physically accurate representation of the behaviour of the CFRP tendon under sustained loading.

Overall, the TML foil gauges are the only strain measurement system which performed consistently and accurately across all three tests in Series B. Further testing is needed to support this evidence, especially over longer durations and at high load ratios. On the other hand, the strain measurements recorded by the three HBK systems were deemed inaccurate and these measurements are hence discarded from further analyses in this study. These results do not, however, undermine the accuracy of HBK strain gauges for sustained loading tests at low load ratios, as reported elsewhere [9]. These combined findings question the existence of a sustained load threshold for the reliability of bonded HBK foil gauges to measure creep strains on CFRP tendons under sustained loading.

It should be noted that during loading, all foil gauges used in this study performed identically to each other and to the other strain measuring techniques used, which highlights the specificity of sustained stress testing compared to conventional quasi-static tensile testing.

**Figure 9:**
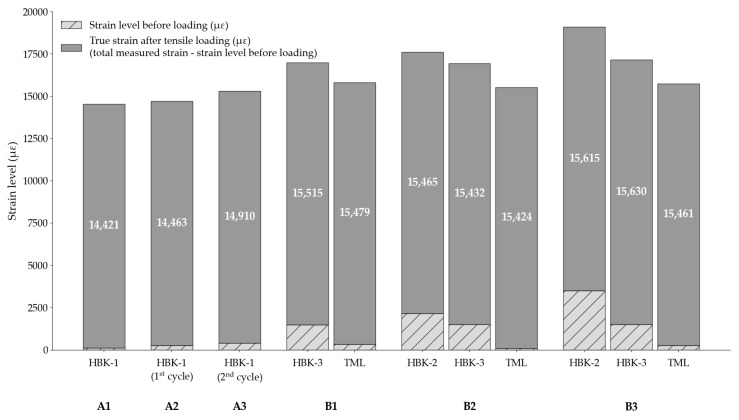
Strain levels recorded by the bonded foil gauges before and after tensile loading.

In addition, the influence of residual strains from the application processes on the behaviour of the foil gauges is assessed in Figure 9. Indeed, it was observed that the bonded foil gauges already recorded a certain level of strain after being connected to the data acquisition system, prior to the start of the loading. As such, the reported relative strain data from the foil gauges in Figure 7 and Figure 8, i.e., the total measured strain minus the initial strain level before loading, are in practice higher, as seen in Figure 9. In Test Series A, the residual strains after application are insignificant for the HBK-1 foil gauges. In Test Series B however, despite using the same application process, the HBK-2 and HBK-3 foil gauges developed high residual strains during the application stage. For example, in Test B3, HBK-2 had already achieved 18% of its maximum strain before loading had started. After the tensile loading, the HBK-2 foil gauge was hence recording a total strain of 19,103 με in practice. Meanwhile, the maximum residual strain for a TML gauge was 329 με in Test B1. The values for true strain after tensile loading, which is defined as the total measured strain minus the strain level prior to loading, recorded by the TML gauges, are consistent for the three tests from Series B. On the other hand, the true strain levels computed for the HBK-2 and HBK-3 foil gauges are generally higher after tensile loading, especially in Test B3, when compared to the true strain levels computed for the TML foil gauge. The presence of high residual strains in the HBK-2 and HBK-3 foil gauges might partly explain the behaviour of these foil gauges during the sustained loading phase of the tests: the residual strains present in the bonded HBK foil gauges might experience a gradual relaxation during sustained loading, leading to an apparent decrease or underestimation of the true creep strains of the CFRP tendon.

In general, once again, the TML foil gauges performed better than the three HBK systems in terms of their sensitivity to developing residual strains during application to the surface of the CFRP tendon. This resilience to application processes strengthens the reliability and reproducibility performance of TML foil gauges under sustained loading conditions. A controlled and meticulous application process, which minimises residual strains, is thus a pre-requisite for reliable foil gauge performance under sustained loading.

### 3.3. Temperature Sensitivities

**Figure 10:**
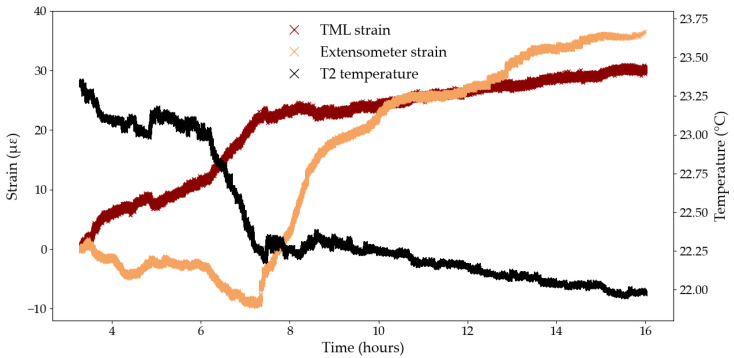
Temperature and strain data in Test B2 between the 3rd and 16th hour of testing.

Another source of variability in the strain data stems from the sensitivity of the sample, the strain sensors, the loading frame, and the system electronics to changes in temperature that can occur during long-term testing. Figure 10 shows the temperature and strain evolution during Test B2, for the extensometer and the TML foil gauge, between the 3rd and 16th hour of testing. Over this time period, the T2 temperature sensor recorded a decrease in temperature of 1.3 °C. The extensometer and the TML gauge both recorded an overall increase in strain, 36 and 30 με, respectively (i.e., the difference between the strain values at the start and end points), which represents approximately 30% of the overall creep strain measured at the end of the test for both strain measuring techniques. The apparent negative coefficient of thermal expansion may indicate that the tendon is expanding as the temperature decreases, which agrees with the CTE of the CFRP composite presented in Section 2.1. The TML foil gauge and the extensometer did not, however, react to the change in temperature in the same manner. Indeed, the extensometer first recorded a decrease of 10 με between the 3rd and the 7th hour of testing, followed by an increase of 46 με in the next 9 h. Meanwhile the TML foil gauge recorded a sharp increase in strain of 24 με between the 3rd and the 7th hour of testing, followed by a more gradual increase of 6 με in the next 9 h. A lag in response to the change in temperature from the TML foil gauge or the extensometer may partly explain this difference in behaviour, although this is challenging to accurately quantify.

**Figure 11:**
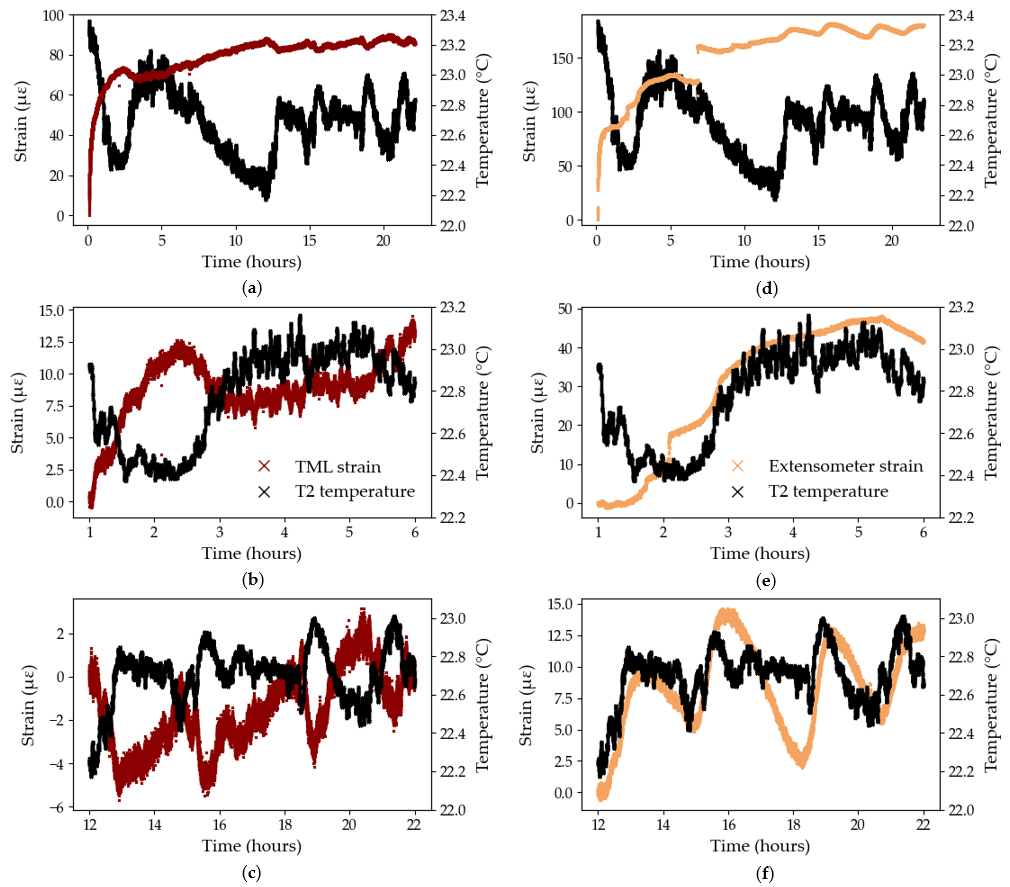
Temperature sensitivities, Test B3: (**a**) TML foil gauge—full test duration; (**b**) TML foil gauge—zoom between the 1st and 6th hour; (**c**) TML foil gauge—zoom between the 12th and the 22nd hour; (**d**) Extensometer—full test duration; (**e**) extensometer—zoom between the 1st and 6th hour; (**f**) extensometer—zoom between the 12th and the 22nd hour.

The results from Test B3 are presented in Figure 11 for the extensometer and the TML foil gauge, for the overall test duration (Figure 11a,d), between the 1st and 6th hour of testing (Figure 11b,e), and between the 12th and 22nd hour of testing (Figure 11c,f), to zoom on noteworthy behaviours. During the first 6 h of testing, the extensometer records a constant, albeit not smooth, increase in strain (Figure 11c) while the strains recorded by the TML gauge appear to mirror the temperature curve both in phase and magnitude (Figure 11b). In Figure 11c,f, the T2 temperature detector recorded periodic 2-hour temperature cycles which were attributed to the ventilation cycles in the testing laboratory at Empa. Similarly to the previous time range, the TML foil gauge seems to capture temperature variations accurately and consistently while the extensometer exhibits a less defined temperature sensitivity, with variability both in phase lag and magnitude. In addition, the zoomed in graphs highlight the wider range of temperature-induced strains recorded by the extensometer, compared to the strains recorded by the bonded TML gauge, thus underlining the apparent greater temperature sensitivity of the contact extensometer compared to the bonded TML gauge. For example, over the first 6 h of testing (Figure 11b,e), for the same temperature profile, the strains recorded by the TML gauge and the extensometer spanned a range of 14 and 48 με, respectively.

**Figure 12:**
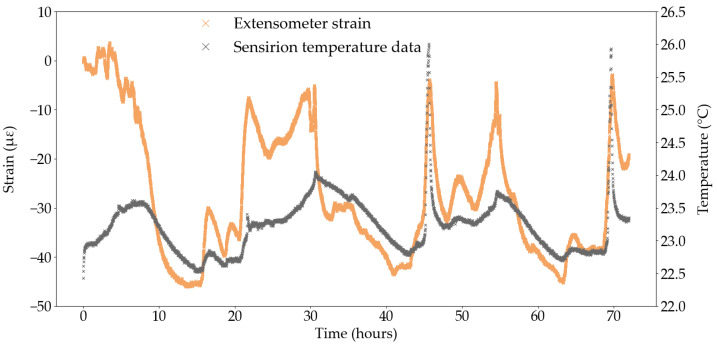
Unloaded test with extensometer strain measurement and temperature data.

To better assess the temperature sensitivity of the extensometer, and remove potential thermal effects induced by loading, a dummy test was conducted on one CFRP tendon from the same manufacturing batch as the tendons used in Series A and B, loaded at 1 kN for 72 h. The strain was measured by the same contact extensometer as in previous tests, using the same testing parameters, and the temperature was recorded by the same Sensirion sensor. As shown in Figure 12, the strain measured by the extensometer follows the general variations in temperature however a phase lag is still present. Figure 12 also confirms the great sensitivity of the extensometer to changes in temperature. For example, in the first 16 h of testing, the absolute temperature variation is 0 °C (i.e., the difference between the strain values at the start and end points) but the extensometer records a decrease in strain of 45 με.

Figure 11c,f can also be used to compute an approximate CTE value for the TML foil gauge and the extensometer. The change in temperature was computed between several points in time corresponding to peaks or troughs in the temperature data and the corresponding change in strain, measured by the foil gauge and the extensometer, was calculated between said points in time. In Figure 11f, the lag in response of the extensometer was accounted for by selecting the anticipated peak or trough in the strain curve which corresponded to that of the temperature curve: this methodology is approximate but serves as a good first estimate of the CTE. As such, the CTE of the TML foil gauge was determined to be −11.54 με/°C with a coefficient of variation of 8%. The negative value likely indicates that the CFRP tendon contracts during an increase in temperature; however, the calculated negative CTE contradicts the positive CTE of the TML foil gauge as reported by the manufacturer (Table 1), despite both values differing by only 2% in magnitude. The CTE of the extensometer was determined as 15.73 με/°C with a coefficient of variation of 22% which attests of the difficulty to quantify the response lag of the extensometer to temperature changes.

An extensometer CTE was also determined from Figure 12 using the same methodology. The extensometer CTE in the unloaded test was determined as 25.33 με/°C with a coefficient of variation of over 59%, which highlights the inadequacy of this approximation method to quantify the sensitivity of the extensometer to temperature changes and questions the validity of a single, constant CTE value to define the temperature sensitivity of the extensometer. In that capacity, a standard CTE for the extensometer is not available from the manufacturer, which is likely due to the wide range of temperature sensitive equipment within the extensometer itself, such as the metal clamps holding the tendon and the instrumentation inside the sensor arms. Moreover, any wiring and electronics connecting the strain sensor to the acquisition system, may respond to a change in temperature. Each component of the strain sensing system has a specific CTE, and a specific response time to temperature stimuli which creates a complex interaction network. This makes it impossible to perform the analytical task to accurately remove temperature effects from the strain signal measured by the extensometer during testing.

The DIC system is another complex strain sensing system with various components sensitive to temperature such as the internal processor, the image sensor, or the battery, which may heat up, particularly over longer testing durations. Figure 6 showed that the camera temperature, in Tests B2 and B3, was the calibration parameter with the highest degree of variability before and after testing, on average between both tests. The camera temperature ranged between 38.20 °C and 39.05 °C which is significantly higher than the temperatures recorded by the T2 temperature detector in Tests B2 and B3, as shown in Figure 10 and Figure 11. The definition of a CTE for the DIC cameras is, however, challenging, given that the DIC strain data is too noisy to allow for typical temperature-induced strain patterns to be visible, as seen in Figure 7 and Figure 8. Moreover, due to the unforeseen interruptions in data acquisition and the low capture frequency, the DIC signal is not sufficiently continuous to distinguish temperature-induced strains. In the same way, the data from the DFOS system (Figure 3) are acquired too sporadically to show any significant variability, including temperature-induced variability.

The temperature sensitivity study highlights the importance of quantifying strains induced by changes in temperature during long-term creep testing of CFRP tendons, before any smoothing is applied to the data. The variability and, often, inconsistency in estimates of CTE for the various strain sensors emphasises the complexity of long-term strain sensing: everything in the test set-up, from the material to the acquisition system, may be sensitive to temperature fluctuations. It is recommended to accurately measure (and ideally control) the temperature during long-term sustained loading tests on CFRP tendons and determine an acceptable, test specific, magnitude of temperature-induced noise. It is recommended that temperature-induced strains developed during the testing should not exceed the overall strains developed at the end of the test. More importantly, selecting strain sensors with a defined CTE (unlike the extensometer or the DIC) which is compatible with the CTE of the CFRP tendons, is essential to accurately compensate temperature-induced strain variations, whether in a controlled laboratory environment, where changes in temperature are small, or in an outdoor environment, where the magnitude and frequency of temperature-induced strain variations will likely increase. In light of the low magnitude of temperature-induced strains quantified in the current study, it is assumed that all temperature-induced effects developed during testing are recoverable. As such, if the temperatures recorded at the start and at the end of the test are in an adequately close range, as defined by the user (a 1 °C range is used in this study), it is deemed acceptable to treat temperature-induced strains as noise that can be smoothed out from the overall measured strains, with an appropriate function or a reliable estimate of the CTE for the strain sensing technique.

### 3.4. Material Inhomogeneities

#### 3.4.1. Distributed Fibre Optic

**Figure 13:**
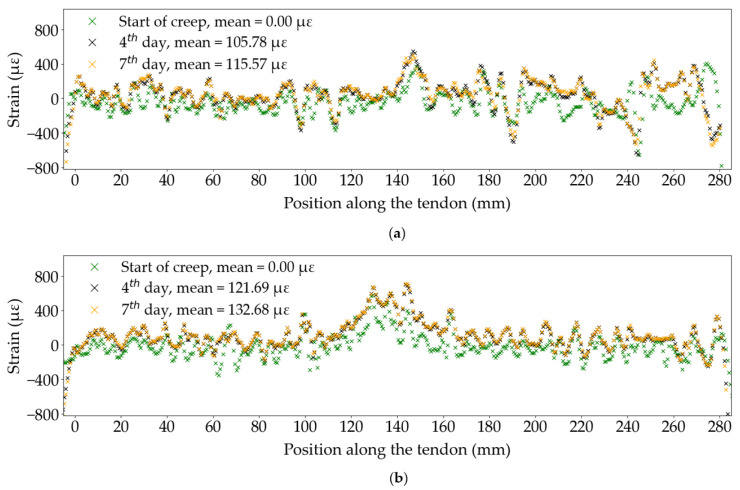
DFOS strain along the free length of the tendon: (**a**) Test A1; (**b**) Test A3.

Distributed fibre optic sensors provide the highest spatial resolution out of all the strain sensing methods presented in this study. Each fibre optic spans 280 mm of the tendon’s surface, which is over 60% of its free length, between the anchors, with a measurement recorded every 0.65 mm. The optical fibre is thus able to capture localised deformations or inhomogeneities on the material’s surface, such as fibre debonding. These defects originate from the pultrusion process or the sustained loading. Figure 13 shows the distribution of strain along the tendon at three points in time, during Tests A1 and A3. The strain is zeroed from the start of the sustained loading phase. All six curves presented in this figure show a considerable variability. In Test A1, several localised drops in strain can be observed, the largest occurring at 245 mm, where the strain drops by about 700 με. Test A3 shows less pronounced peaks in strain but continuous ±200 με variability. In both tests, a concentration in strain of approximately 600 με can be seen at the midpoint on the tendon’s length (150 mm in Test A1, 130 mm in Test A3), which was seen to develop during the loading phase and might be caused by concentrated bending of the tendon at its mid length, due to imperfect alignment of the test specimen in the loading frame. However, it is impossible to accurately determine whether a peak in strain data measured by the DFOS represents a system-induced strain, which should be removed during post-processing, or depicts a natural inhomogeneity on the material’s surface. The average for each curve in Figure 13 was thus computed to include all data points between 10 mm and 270 mm on the tendon’s length to reduce boundary effects at the edges of the bonded fibre. Despite the apparent variability in strain along the tendon, the computed strain averages for all positions, at each point in time, lead to smooth strain profiles, as seen earlier in Figure 3.

In these specific tests, the variability in strain data captured by the DFOS along the tendon’s free length does not appear to influence the overall creep strains of the CFRP tendon at any point in time. As such, the insights from DFOS strain data can help validate the robustness of the test set-up. It is nonetheless essential to examine the longitudinal distribution of strain data points from DFOS to distinguish natural material inhomogeneities from sensor-induced variability, such as debonding of the optical fibre from the tendon’s surface, inhomogeneities in the thickness of the adhesive between the fibre and the tendon, excessive bends in the optical fibre (minimal issue in the case of straight tendons in tension), and testing set-up failures such as excessive bending of the tendon in the loading frame. This discretion must be material and test-specific and educated decisions should be made to validate the distributed strain data before processing. This observation reinforces the importance of continuous measurements to capture sudden system failures, which is unfortunately not possible with DFOS systems due to the limited service life of the laser source (typically 25,000 h for a LUNA ODiSI DFOS as used in the current study).

#### 3.4.2. DIC Gauge Length

**Figure 14:**
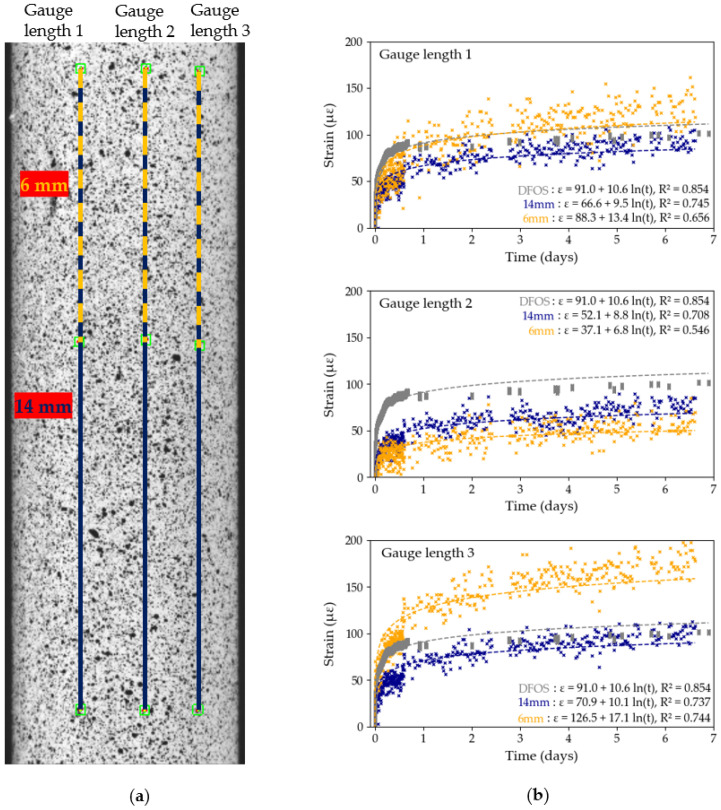
Test A1, DIC gauge length sensitivity study: (**a**) position of gauge lengths on the ROI; (**b**) strain recordings over time for full (14 mm) and short (6 mm) gauge lengths with logarithmic trendlines. The lack of DIC data between the second and the third day corresponds to an interruption in the DIC image capture system.

**Figure 15:**
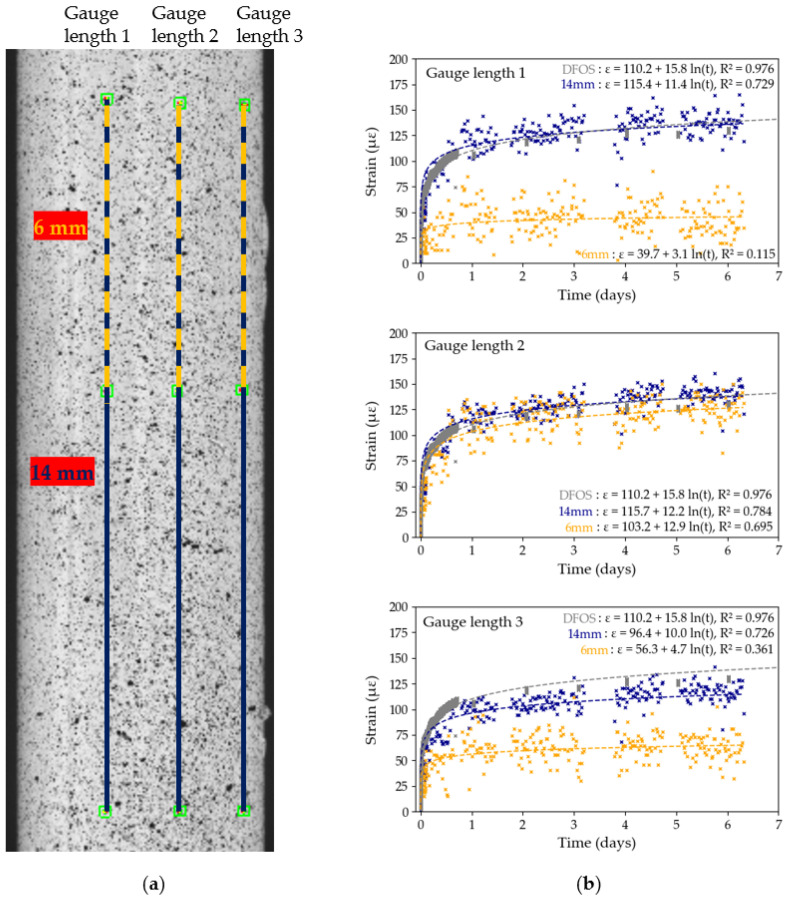
Test A3, DIC gauge length sensitivity study: (**a**) position of gauge lengths on the ROI; (**b**) strain recordings over time for full (14 mm) and short (6 mm) gauge lengths with logarithmic trendlines. The lack of DIC data at the end of the first day, between the third and the fourth days and at the end of the fourth day corresponds to an interruption in the DIC image capture system.

DIC provides another approach for assessing the variability in strain along the tendon’s free length by varying the length of the gauge used to compute the strains. In particular, two gauge lengths are studied and presented in Figure 14 and Figure 15, for Tests A1 and A3, respectively: a 14 mm long gauge spanning the full ROI and a 6 mm long gauge length, to mimic the length captured by the foil gauges used in this study. In addition, three gauge positions were selected to assess the variability in measured strains across the tendon’s surface, as previously discussed in Section 3.1.2. In these two tests, calibration parameters were only obtained after the end of the test. Logarithmic curves were fitted to the data using Python’s curve-fitting function; the curve equation and $R^{2}$ value are provided for each data set. In both figures, the trendlines fitted to the DFOS data were also added to validate the DIC data.

In all six cases, except gauge 3 from Test A1, the $R^{2}$ value for the full gauge length is higher than that of the short gauge length, in some cases significantly higher: in Test A3, the long gauge length 1 has an $R^{2}$ value over six times higher compared to the shorter gauge length. Strain measurements obtained by DIC are hence more precise with the longer gauge lengths as the effects of surface inhomogeneities on the overall creep strain of the tendon seem to be reduced. This observation is validated by comparing the two gauge length data sets with the data obtained by DFOS: in general, the DFOS trendline is closer to the 14 mm gauge length trendline, especially in Test A3 (Figure 15). On the other hand, the 6 mm gauge lengths, which are representative of a bonded foil gauge length, captured noisy and inconsistent data. For example, in Test A3, the maximum strains captured by the short gauge lengths spanned 83 με across the three gauge lengths, against 23 με for the longer gauge lengths. This observation may also explain the significant level of noise captured by the HBK-1 foil gauges in Figure 3.

Moreover, the strain data captured by the longer gauge lengths is consistent across the three positions on the CFRP tendon, in both Figure 14 and Figure 15. In Section 3.1.2, however, Figure 7 and Figure 8 highlighted a significant variability in computed strains across the tendon’s diameter in Tests B2 and B3, despite the nominal gauge length being longer than in Tests A1 and A3. The variability in strain data across the curved surface of the CFRP tendon and the highlighted inconsistencies between tests warrants a meticulous cross-validation of the DIC results before any analysis can take place.

In addition, the $R^{2}$ values in Tests A (Figure 14 and Figure 15) are generally lower compared to the $R^{2}$ values in Tests B (Figure 7 and Figure 8), which may be caused by the quality of the original speckle pattern or by the increased duration of Tests A (7 days versus 22 h for Tests B). Prolonged high loading may induce cracks in the paint or delamination of fibres on the tendon’s surface, as captured by the second camera in Figure 8a, which, over time, may impact the quality of the acquired strain data. These findings question the suitability of DIC for measurements taken over more than seven days.

## 4. Discussion: Creep of CFRP Tendons

The prior Section 3 described the main sources of variability in long-term creep measurements of CFRP tendons and quantified their influence on the strain data measured by the multiple strain sensing techniques. In general, all strain sensing techniques, except HBK foil gauges, were able to measure creep strains of the CFRP tendon in the range of 0.03%. The strain sensing techniques all had their advantages and disadvantages and varying degrees of sensitivity to the identified sources of variability. As such, the challenge of accurately quantifying creep strains of CFRP tendons depends less on the choice of strain sensing technique but more on validation of experimental strain data through rigorous data processing.

In light of the previous observations, measurements from the cross-validated strain measuring techniques were selected from the two Test Series, and creep coefficients, $C_{c}$, were computed as percentages:

(3) $$C_{c}=\frac{\varepsilon_{C}}{\varepsilon_{E}}100$$

where $\varepsilon_{C}$ is the achieved creep strain at the end of the test and $\varepsilon_{E}$ is the initial elastic strain. The results are summarised in Table 4. All HBK foil gauges were discarded considering the results from Section 3.2. For the DIC data analysis, the data set with the highest $R^{2}$ value in each test was selected for further analysis. As such, in Test A1, 14 mm long gauge 1 was chosen (see Figure 14); in Test A3, 14 mm long Gauge 2 was chosen (see Figure 15); in Test B2, Gauge 3 and pre-test calibration parameters were used (see Figure 7); and in Test B3, Gauge 2 and pre-test calibration parameters were used (see Figure 8). For the two extensometer data sets, the jumps in strain were removed during post-processing as they were shown to originate from movement of the test set-up in Section 3.1.1. Trendlines were obtained with Python’s logarithmic curve-fitting function enabling 50 year creep coefficient predictions. The creep coefficients determined in Tests A and B are in the same order of magnitude as creep coefficients recorded in the literature, despite the current tests being significantly shorter than conventional creep tests: Ascione et al. [5] found a creep coefficient of 1.93 after 76 days for pultruded CFRP laminates loaded at 75% of UTS from bonded strain gauge measurements; Yang et al. [8] found a creep coefficient of 0.95 after 1000 h for pultruded CFRP tendons loaded at 85% of UTS from a custom pressure dial strain measuring device. Within the same test, a variability remains between the creep coefficients of different strain measuring devices. For example, in Test B3, the measured creep coefficient varies by 70% between DIC and extensometer measurements, a difference which is reduced to 5% between the 50 year-predicted creep coefficients. As such, despite efforts to minimise the sources of data variability during testing and a meticulous first screening of the strain data from all the strain measuring techniques in this study, discrepancies remain, which reinforces the necessity to assess the reliability of strain data in creep experiments.

It is important to note, however, that despite validation of the test results presented in Table 4, the insufficient number of successful repeats in each test does not permit conclusive remarks on the behaviour in creep of the CFRP tendons tested in this study.

To validate the predicted 50-year creep coefficients in Table 4, the 50-year elastic modulus can be computed based on the rule of mixtures. Given the elastic moduli of the matrix and the fibres, $E_{M}$ and $E_{F}$, respectively, presented in Section 2.1, and a fibre volume fraction $\Phi_{F}$ of 72%, the theoretical elastic modulus of the unidirectional CFRP tendon, $E_{11}$, in the direction of the fibres and assuming perfectly parallel carbon fibres, which is not the case in practice with pultrusion processes, can be computed as follows:

(4) $$E_{11}=E_{F}\Phi_{F}+E_{M}\left(1-\Phi_{F}\right)$$

to give a value of 166,580 MPa. After 50 years, it can conservatively be assumed that the contribution of the epoxy to the elastic modulus of the CFRP tendon becomes negligible due to the sustained creep. As such, the 50-year assumed elastic modulus $E_{50}$ of the CFRP tendon becomes 165,600 MPa, i.e., a decrease of 0.588%. Equation (3) can be rearranged to calculate the predicted theoretical creep coefficient after 50 years:

(5) $$C_{c}=\frac{E_{11}-E_{50}}{E_{50}}100$$

which gives a value of 0.592%. Despite a similitude with the predicted values in Table 4, which validates the experimental results in this study, the theoretical 50-year creep coefficient is lower than the predicted 50-year creep coefficients in Table 4, which indicates that creep of the epoxy matrix may not be the only source of increase in strain of the CFRP tendon under sustained loading.

Nevertheless, the creep coefficient values reported in Table 4 are small and confirm the excellent performance of unidirectional pultruded CFRP bridge tendons under sustained loads. At most, a 50 year creep coefficient of 2.17% can be predicted from DFOS data during a 7 day long creep test, which raises questions as to the validity (and potential over-conservatism) of the current restrictive design guidelines, thus strengthening the need for additional reliable long-term creep testing data for CFRP tendons.

## 5. Conclusions

In this study, movement of the test set-up, temperature sensitivity of the testing equipment, and localised inhomogeneity of the sample’s surface were identified as the three main sources of variability in experimental creep strain data of pultruded CFRP tendons. The tendons were instrumented with bonded foil strain gauges, a contact extensometer, a DIC system, and adhesively bonded DFOS to capture strain data in the range of 0.03% under sustained tensile loading at 80% (for 7 days) and 88% (for 22 h) of their UTS. A comparative analysis of the performance of each strain sensing technique revealed distinct sensitivities to the highlighted variability sources:

- The contact extensometer recorded jumps of up to 250% in measured strain due to sudden movement of the anchored CFRP tendon during sustained loading, while bonded strain sensing techniques were unaffected by movement of the test set-up.
- The bonded TML strain gauge recorded a small and recoverable range of temperature-induced strains, consistently 12 με/°C, while the response of the extensometer to temperature changes varied in phase and magnitude, with a maximum reported CTE of 40 με/°C.
- The stereoscopic DIC set-up was shown to be highly sensitive to curved surfaces and out-of-plane motions with a variability of up to 43% in computed creep strains between different gauge length positions across the tendon’s diameter.
- The DFOS recorded localised peaks of 150% of the averaged measured strain at the tendon’s mid length; however, these localised strain peaks had no significant influence on the overall creep behaviour of the CFRP tendons.
- The HBK foil gauges either recorded a decrease in strain over time, which disagrees with the physical definition of creep, or underestimated the creep strains when compared to the other strain sensing techniques, demonstrating their inadequacy for measuring strain of CFRP tendons under high sustained loading conditions.

The magnitude of test- and sensor-induced noise in the measured creep strain data emphasises the necessity of rigorous experimental precautions, analytical awareness, and research methods transparency when testing CFRP tendons for creep. Following cross-validation of the measured strain data, a maximum creep coefficient of 2.17% was predicted after 50 years at 88% of UTS for the unidirectional pultruded carbon-epoxy CFRP tendons used. However, further long-term reliable creep data are necessary to comprehensively assess the creep behaviour of CFRP tendons and challenge the current conservative design guidelines.

## Figures and Tables

**Table 1:** Specifications of adhesively bonded foil gauges used in this study.

Foil Gauge	Dimensions (mm) ^†^	Resistance (Ω)	Gauge Factor	Rated Strain Limit	CTE * ($10^{-6}$/°C)
HBK 1-LY5x-6	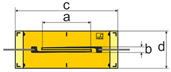 [18] a=6.0, b=0.4 c=13.0, d=4.7	120	2.06 ± 1.0%	5%	10.8
HBK 1-LY4x-6	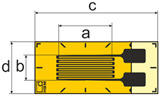 [18] a=6.0, b=2.8 c=13.9, d=5.9	350	2.09 ± 1.0%	5%	10.8
TML FLKB-6-11	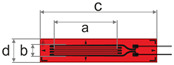 [17] a=6.0, b=1.0 c=11.0, d=2.2	120	2.10 ± 1.0%	5%	11.8

^†^ Images are not to scale. * CTE: Coefficient of thermal expansion (measured on steel).

**Table 2:** Four foil gauge systems.

System Name	Foil Gauge	Adhesive
HBK-1	HBK 1-LY4x-6	Z70
HBK-2	HBK 1-LY5x-6	X60
HBK-3	HBK 1-LY5x-6	CA80
TML	TML FLKB-6-11	EP2

**Table 3:** Testing matrix.

Test Name	Load Level	Expected Strain Level	Test Duration	Strain Sensors	Other Sensors
Test A1	80%	1.42%	7 days	HBK-1 DFOS DIC	—
Test A2	80%	1.42%	1st cycle: 10 h 2nd cycle: 7 days	HBK-1 DIC	Pi-gauge
Test A3	80%	1.42%	1st cycle: 7 days 2nd cycle: 7 days	HBK-1 DFOS DIC	Pi-gauge
Test B1	88%	1.53%	4.2 h (anchor failure)	HBK-3 TML Extensometer DIC	T1, T2 Sensirion Accelerometer
Test B2	88%	1.53%	22 h	HBK-2, HBK-3 TML Extensometer DIC	T1, T2 Sensirion
Test B3	88%	1.53%	22 h	HBK-2, HBK-3 TML Extensometer DIC	T1, T2 Sensirion Accelerometer

**Table 4:** Creep test results and predictions.

Test Name	Strain Device	Creep Coefficient at End of Test (in %)	Creep Equation (t in Hours)	R^2^ Value	50-Year Creep Coefficient Prediction (in %)
A1	DIC	0.75 (7 days)	66.57 + 9.48 ln(t)	0.745	1.36
	DFOS	0.69 (7 days)	91.04 + 10.56 ln(t)	0.854	1.54
A3	DIC	0.98 (7 days)	115.70 + 12.17 ln(t)	0.784	1.82
	DFOS	0.90 (7 days)	110.17 + 15.76 ln(t)	0.976	2.17
B2	TML foil gauge	0.62 (24 h)	63.49 + 12.92 ln(t)	0.903	1.51
	Extensometer	0.82 (24 h)	51.60 + 12.40 ln(t)	0.616	1.38
	DIC	0.75 (24 h)	65.89 + 15.06 ln(t)	0.950	1.71
B3	TML foil gauge	0.56 (22 h)	58.42 + 9.73 ln(t)	0.918	1.20
	Extensometer	0.32 (22 h)	96.38 + 16.82 ln(t)	0.897	2.03
	DIC	1.05 (22 h)	73.43 + 19.89 ln(t)	0.957	2.14

## Data Availability

The original contributions presented in this study are included in the article. Data are contained within the article. Further inquiries can be directed to the corresponding author.

## Abbreviations

The following abbreviations are used in this manuscript:

CFRP	Carbon Fibre-Reinforced Polymer
DIC	Digital Image Correlation
DFOS	Distributed Fibre Optic System
UTS	Ultimate Tensile Strength
CTE	Coefficient of Thermal Expansion
RTD	Resistance Temperature Detector
ROI	Region of Interest
